# Mobile App/Web Platform for Monitoring Food Oral Immunotherapy in Children: Longitudinal Clinical Validation Study

**DOI:** 10.2196/54163

**Published:** 2024-03-13

**Authors:** Sergio Sánchez-Fernández, Eva María Lasa, Soledad Terrados, Francisco Javier Sola-Martínez, Sara Martínez-Molina, Marta López de Calle, Paula Cabrera-Freitag, María José Goikoetxea

**Affiliations:** 1 Allergy and Clinical Immunology Department Clínica Universidad de Navarra Pamplona Spain; 2 Pediatric Allergy Unit Allergy Service Hospital Universitario Donostia Donostia-San Sebastián Spain; 3 Department of Pediatric Allergy Ramón y Cajal Hospital Madrid Spain; 4 Pediatric Allergy Unit, Allergy Department Hospital General Universitario Gregorio Marañón Madrid Spain; 5 Gregorio Marañón Health Research Institute Madrid Spain; 6 Instituto de Investigación Sanitaria de Navarra Pamplona Spain; 7 RICORS Red De Enfermedades Inflamatorias - RD21/0002/0028 Madrid Spain

**Keywords:** adverse reactions, egg allergy, food oral immunotherapy, mHealth, milk allergy, monitoring

## Abstract

**Background:**

Milk and egg allergies significantly impact the quality of life, particularly in children. In this regard, food oral immunotherapy (OIT) has emerged as an effective treatment option; however, the occurrence of frequent adverse reactions poses a challenge, necessitating close monitoring during treatment.

**Objective:**

This study aims to evaluate the ability of a new mobile/web app called OITcontrol to monitor milk and egg OIT.

**Methods:**

Patients undergoing milk or egg OIT were recruited and divided into 2 groups: the active group used the OITcontrol app in conjunction with standard written monitoring methods, whereas the control group relied solely on written diaries. Investigators documented hospital doses, hospital reactions, and administered treatments on the website. Patients recorded their daily allergen home-dose intake, home reactions, and administered treatments using the app. The following variables were compared between both groups: number and severity of hospital and reported home reactions, patient’s adhesion to the OITcontrol app or written diary or both in terms of daily home-dose intake and home reactions recording, and treatment and dose adjustment compliance at home in case of reaction.

**Results:**

Sixteen patients were assigned to be monitored using the OITcontrol app along with additional written methods (active group), while 14 patients relied solely on a written paper diary (control group). A similar distribution was observed in terms of sex, age, basal characteristics, allergen treated in OIT, premedication, and sensitization profile. Active patients reported a comparable number of hospital and home reactions compared with the control group. In terms of recording system usage, 13/16 (81%) active patients used the OITcontrol app, while 10/14 (71%) control patients relied on the written diary. Among active patients, 6/16 (38%) used both methods, and 1 active patient used only written methods. However, control patients recorded home reactions more frequently than active patients (*P*=.009). Among active patients, the app was the preferred method for recording reactions (59/86, 69%), compared with the written diary (15/86, 17%) or both methods (12/86, 14%; *P*<.001). Treatment compliance in home-recorded reactions was similar between both groups (*P*=.15). However, treatment indications after an adverse reaction were more frequently followed (*P*=.04) in reactions recorded solely in the app (36/59, 61%) than in the written diary (29/71, 41%) or both systems (4/12, 33%). Moreover, compliance with dose adjustments after a moderate-severe reaction in home-recorded reactions was higher in the active group than in the control group (*P*<.001). Home reactions recorded only in the app (16/19, 84%) were more likely to follow dose adjustments (*P*<.001) than those recorded in the written diary (3/20, 15%) or using both methods (2/3, 67%).

**Conclusions:**

The OITcontrol app appears to be a valuable tool for monitoring OIT treatment in children with food allergies. It proves to be a suitable method for recording daily home dose intakes and reactions, and it seems to enhance adherence to treatment indications following an adverse reaction as well as compliance with dose adjustments in home reactions. However, additional studies are necessary to comprehensively grasp the benefits and limitations of using the OITcontrol app in the management of OIT.

## Introduction

Food allergies are increasingly prevalent worldwide, particularly within the European population [1]. Children, in particular, often experience allergies to common foods such as eggs and milk, which can lead to severe reactions [2]. Notably, these allergies constitute a significant factor in causing anaphylaxis during early childhood [3]. Research indicates that approximately 50% of children with allergies naturally outgrow their milk and egg allergies. However, a substantial number of patients do not experience spontaneous resolution of these allergies [4,5]. The prevailing method for treating food allergies involves the complete avoidance of the allergen. However, with milk and egg allergies, which are found in numerous everyday foods, steering clear of the allergen is challenging. Consequently, over 20% of children and adolescents who experience anaphylaxis are already aware of the allergen, necessitating avoidance [3]. Indeed, food allergies in children significantly impact the quality of life for parents and caregivers, particularly in terms of the self-management of the condition [6].

Oral immunotherapy (OIT) for food, involving the oral administration of allergens to induce tolerance, has proven to be an effective treatment for persistent food allergies in children, despite the occurrence of frequent adverse reactions [7-9]. In typical OIT protocols, incremental doses of the allergenic food are administered in a hospital setting, and once tolerated, these doses are continued daily at home. This daily allergen consumption continues until the target food dose is reached, marking the completion of the buildup phase [10]. Subsequently, the established target dose is maintained at home to sustain the acquired tolerance, marking the beginning of the maintenance phase. Although most reactions typically occur during the hospital-based buildup phase, it is noteworthy that reactions can also manifest during the maintenance phase, after home dose intake [11,12]. Patients undergo education on avoiding potential cofactors and managing potential reactions at home [13]. Furthermore, it is crucial to adjust the prescribed allergen dose in the event of a reaction [14] or if patients are experiencing an intercurrent disease [10]. This information, along with specific treatment guidelines for addressing reactions at home, is typically conveyed verbally and in writing to caregivers and patients undergoing OIT treatment. Indeed, the management of OIT necessitates vigilant oversight both from the medical staff during hospital-based doses and from patients and their families during home-based doses.

Certain studies have reported an enhancement in the quality of life for patients treated with OIT at the culmination of the buildup phase [15-19]. However, contrasting findings exist, with some studies demonstrating no discernible differences [20] and others even describing a decline in the quality of life for certain patients following OIT treatment [21]. It has been suggested that the absence of improvement in quality of life after OIT could be linked to the numerous hospital visits required during the up-dosing phase [22] and the frequent occurrence of adverse reactions [23].

To assist patients in managing home doses and provide targeted information in conjunction with OIT treatment, a web platform designed for health staff and a hybrid mobile app for patients, named OITcontrol (University of Navarra, Pamplona, Spain), have been developed. OITcontrol enables medical staff to record doses and reactions in the hospital, and caregivers/patients can use it to log information regarding doses and reactions while at home. OITcontrol serves as a reminder for the timing and administration instructions for daily home doses. Additionally, it provides guidance on specific treatments following a reaction and offers evidence-based dose adjustment instructions through dedicated algorithms [24-26].

The objective of this study was to assess the effectiveness of the OITcontrol app in monitoring patients undergoing food OIT treatment, with a focus on (1) evaluating its capability to document adverse reactions occurring at home, and (2) examining patient adherence to specific recommendations regarding home adverse reactions, including prescribed treatment and adjustments for the next day’s dose.

## Methods

### Study Population

This study was conducted in Spain, specifically at the Hospital Universitario Donostia in Donostia-San Sebastián and Hospital Ramón y Cajal in Madrid. The participants were patients aged either 2 years and older for those diagnosed with milk allergy or between 5 and 18 years old for those diagnosed with egg allergy. The diagnosis was established through immunoglobulin E (IgE)–derived clinical history and positive skin prick tests, IgE sensitization to the allergenic food, or both. These patients were invited to undergo OIT treatment in accordance with the Spanish OIT guidelines [10], with the study period spanning from April 2019 to April 2021. Parents of patients or their legally authorized representatives, and in the case of a mature minor, the children themselves, were provided with comprehensive information regarding the risks and benefits associated with the OIT treatment. Those patients who opted for OIT and reported the use of smartphones were extended an invitation to participate in the study. The participants were monitored until they completed the OIT buildup phase or until the predetermined conclusion of the study in April 2021.

### Ethics Approval

Before participation, written informed consent was acquired from all involved patients, adhering to the prevailing ethical-legal regulations, as outlined in the Helsinki Declaration. The study protocol received approval from the ethics committees of all participating hospitals (2018.199 University of Navarra; PI2017053, Euskadi; Hospital Universitario Ramón y Cajal).

### Allergy Diagnosis

For patients undergoing milk or egg OIT, a skin prick test was conducted using commercial extracts of milk, alpha-lactalbumin, beta-lactoglobulin, and casein for milk allergy, whereas white and yolk egg, ovomucoid, and ovalbumin were used for evaluating egg allergy. Measurements of wheal and flare sizes were taken 15 minutes after the test, and wheals with a diameter equal to or greater than 3 mm were deemed positive [27]. The determination of specific IgE levels for the entire extract (milk or white and yolk egg) and its components (alpha-lactalbumin, beta-lactoglobulin, and casein for milk or ovomucoid and ovalbumin for egg) was conducted using fluorescence enzyme immunoassay with ImmunoCAP (Thermo Fisher). Specific IgE values equal to or exceeding 0.35 kUA/L were classified as positive.

### Food OIT Treatment Protocols

Patients underwent treatment with initially grouped dosing schedules at the hospital, in accordance with the Spanish OIT guidelines [14]. Subsequently, weekly increments in hospital doses were administered. The allergen dose that was tolerated at the hospital was then maintained daily at home between hospital visits. For milk OIT, ultra-high temperature milk was used until, whenever feasible, the final dose of 200 ml milk was reached. For egg OIT, the process involved the use of lyophilized egg white powder (ovo-des; Cantabria Labs Nutrición Médica), pasteurized egg white, or boiled whole egg until the target of 4000 mg of egg white powder, 30 ml of pasteurized liquid egg white, or 1 boiled whole egg was achieved, respectively, where possible [14].

### Intervention

**Figure 1:**
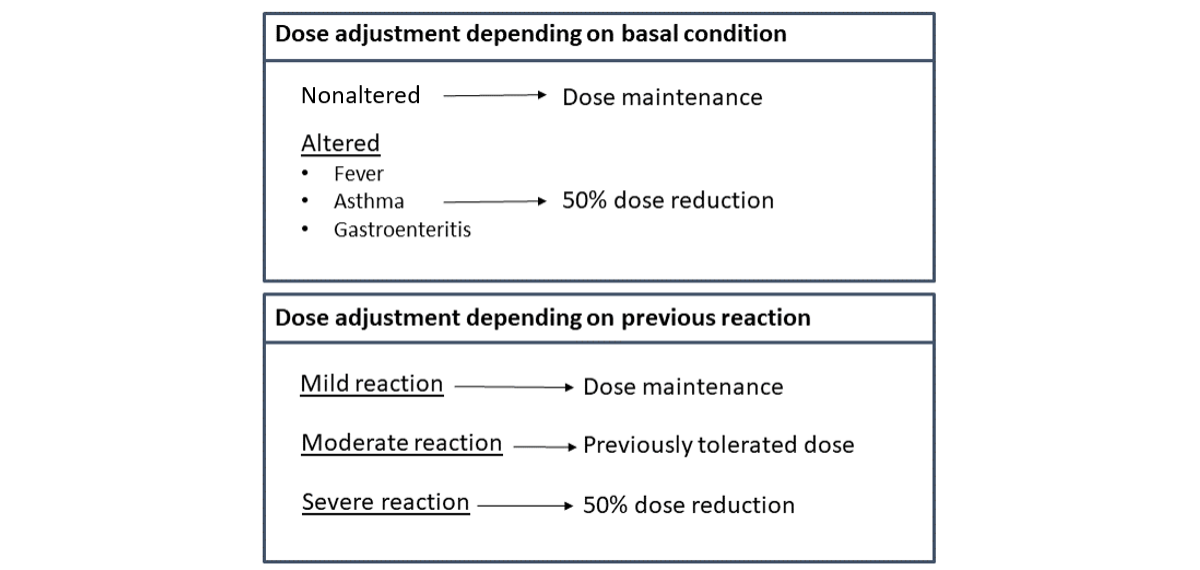
Patient's specific information described in the written diary or implemented in the OITcontrol app.

Before commencing OIT, patients were consecutively recruited, ensuring a balanced distribution between the control group (PaperPRO group) and the active group (OITcontrol group). The medical staff provided oral and written general recommendations to all patients and caregivers (referred to as patients hereafter). All patients were given detailed explanations and written instructions regarding various aspects, including how to administer the allergen dose, a list of cofactors to avoid, guidelines for treating different types of home reactions, and instructions for dose adjustment following a moderate/severe reaction or in the presence of altered basal conditions (axillary fever of ≥38°C, asthma, or gastroenteritis; Figure 1) [8-10]. Patients underwent training to manage home reactions, which included the use of specific medication tailored to each type of reaction (Table S1 Multimedia Appendix 1) [24]. Furthermore, within the category of severe reactions, written recommendations for patients outlined 2 additional severe reactions: anaphylaxis, which was considered when 2 or more symptoms distinct from oral allergy syndrome (OAS) were reported, and anaphylaxis with bronchospasm, when bronchospasm was one of the symptoms accompanying anaphylaxis. For these scenarios, prescriptions of epinephrine and a combination of epinephrine and bronchodilator (salbutamol) were provided, respectively. The term “anaphylaxis” is used in the “Results” section to describe an anaphylactic reaction, irrespective of the presence of bronchospasm. The severity of reactions was categorized based on Sampson’s severity classification into mild, moderate, and severe reactions [28].

Patients were instructed to maintain a daily record of the allergen dose taken and any reactions experienced, noting the type of reaction and the administered treatment, in a paper-based diary as part of patient-reported outcomes (PaperPRO). Furthermore, individuals in the active group were provided training on the utilization of the OITcontrol app on their smartphones to document home doses and reactions. These patients were also encouraged to concurrently use the written diary (OITcontrol group).

### OITcontrol App

**Figure 2:**
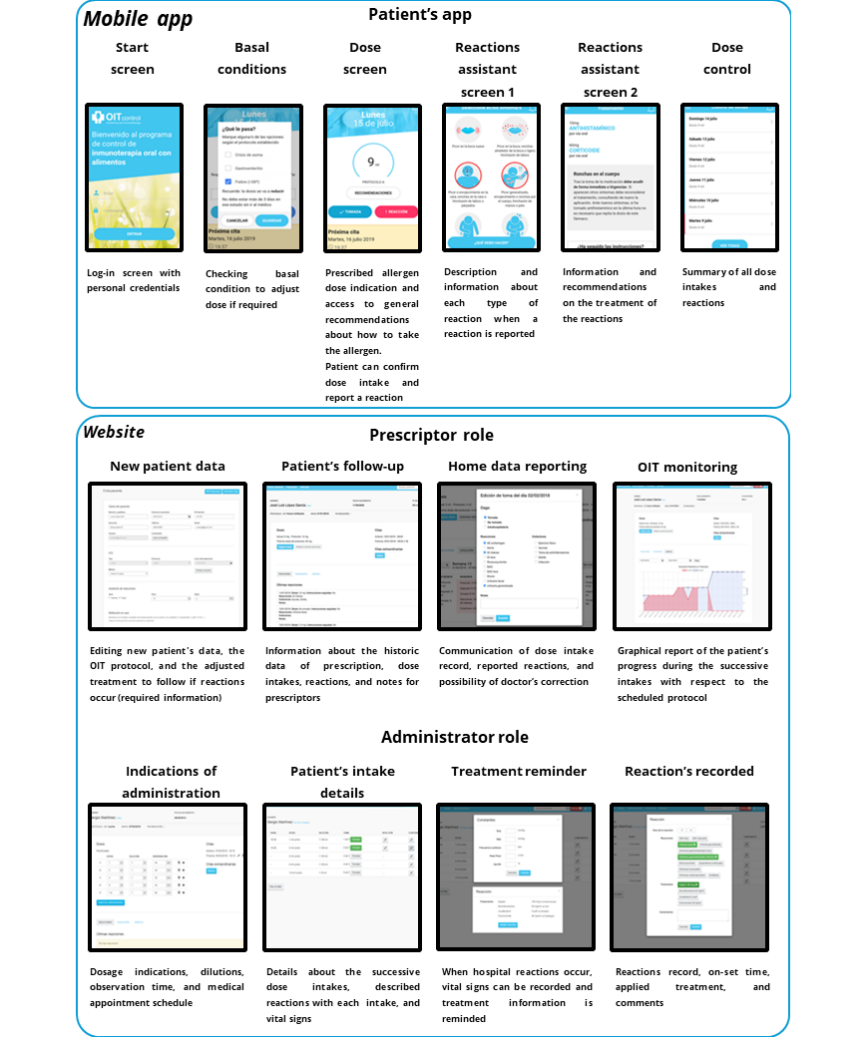
Visual description of the functions and usability of the OITcontrol app for patients and website for medical staff.

OITcontrol is a mobile app designed for patients, available on Google Play (Google LLC) or Apple Store (Apple Inc.) [29], and a website for health staff [30], accessible through 3 distinct user interfaces: (1) The doctor’s interface for prescribing allergen and rescue treatment, accessible as a website platform recommended for use on a computer; (2) the interface for nurses or health personnel responsible for administering food/medication doses, accessible as a website platform intended for use on a tablet; and (3) the patient interface, available as a mobile app, accessible exclusively through the log-in credentials provided by the doctor (Figure 2).

Within OITcontrol, when a doctor prescribes an OIT treatment for a patient, the app allows for the prescription of allergen dose increases, scheduled step rises following the OIT protocol, and outlines home/hospital treatment procedures in the event of a reaction (doctor’s credentials are necessary for access). Once the treatment commences, the app provides daily reminders for the patient’s dose, indicates the observation time, and incorporates an algorithm outlining actions and treatments to be used in the case of a home reaction, contingent on the type of reaction [24] (Table S1 Multimedia Appendix 1). Each symptom is associated with a specific indication in the app. In addition, the app computes 2 additional severe reactions: anaphylaxis, identified when 2 or more symptoms distinct from OAS are reported, and anaphylaxis with bronchospasm, recognized when anaphylaxis occurs alongside symptoms of bronchospasm, mirroring the written recommendations. The app provides general recommendations on how to take the daily dose, including guidance on avoiding cofactors, taking the dose at a consistent time, and the need for observation and rest after dose intake. These recommendations align with those provided in writing to every patient.

The platform/app is designed to retain the last tolerated allergen dose on a daily basis. It does not automatically prescribe increases in allergen dose. However, it is programmed to automatically decrease the dose in 2 specific situations:

- When the basal condition is linked to a reaction, such as in the presence of gastroenteritis, fever, or an asthma attack, the app automatically decreases the dose to half of the scheduled amount [8,10].
- In the event of a moderate/severe reaction, the app adjusts the dose for the next day [8,26] (Figure 1).

OITcontrol facilitates guiding the patient through home treatment and enables medical staff to closely monitor the patient, even when they are at home.

For the health staff, OITcontrol serves several functions:

- It accumulates the complete OIT history of the patient, including protocol modifications, allergen doses linked to reactions, and cofactors involved in reactions. This information is provided by the patient at home and by the health staff during hospital visits. These data are accessible in real-time, constituting an electronic data capture system.
- It serves as an electronic prescription tool for drugs and allergen doses, allowing doctors to prescribe electronically.
- It sends real-time notifications about the patient’s reactions. In the case of a severe reaction, a second notification is dispatched via email.
- It facilitates the management of hospital dose administrations, covering both the multiple-dose initial phase and the unique-dose weekly increase. It generates a summary of the hospital visit, which can be integrated into any digital history system. The app allows for the export of structured text containing patient-specific data for seamless integration.
- It conducts an anonymous analysis of clinical data from hospital patients, considering factors such as the type of reactions, age, sex, and assigned protocol.

### Data Collection

The OIT buildup phase was organized into weekly hospital visits with interhospital home doses. Investigators documented hospital doses, the type of reactions that occurred during hospital visits, and the administered treatment. At home, all patients were encouraged to record daily outcomes in a written diary, including the amount of the daily allergen dose taken, instances of reactions, the type of symptoms experienced, and the treatment administered. Moreover, active patients were encouraged to use the OITcontrol app to document comparable information. The use of the OITcontrol app or a written diary was deemed effective when at least five consecutive home doses had been registered or when 1 reaction had been recorded during the follow-up. The medical staff documented the information in the digital clinical history regarding home dose intakes and home reactions reported verbally by the patients during weekly hospital visits.

### Users’ Satisfaction Questionnaire

An anonymous electronic satisfaction questionnaire regarding the use of the OITcontrol app was distributed to the initial 10 patients in the OITcontrol group. Additionally, 11 previous patients were included in the survey to verify the correct performance and use of the app before initiating the validation study. These participants were recruited from both the Hospital Universitario Donostia in Donostia-San Sebastián and Hospital Ramón y Cajal in Madrid. The same questionnaire was administered after the first and fourth week of app use. The questionnaire included a few demographic questions and inquired about the general impression of the app, the assessment of texts and screens, and the evaluation of terminology. The respondents provided ratings on a scale ranging from 1 (poor) to 5 (good) or from 1 (poor) to 9 (good) for the 22 items included in the questionnaire.

### Statistical Analysis

The distribution of variables was assessed for normality using the Shapiro-Wilk test. Normally distributed quantitative values were presented as mean and SD, while nonnormally distributed quantitative values were described as medians and IQRs (Q1-Q3). Qualitative values were reported as frequencies (percentages). Proportions were compared using the chi-square test or Fisher exact test when the expected frequencies were below 5. Quantitative variables were analyzed using the Student *t* test or Mann-Whitney *U* test based on normality. The clinical statistical analysis was conducted using Stata IC 12.0 (StataCorp LLC). Differences with a *P* value <.05 were considered statistically significant.

## Results

### Baseline Characteristics of the Sample

Thirty participants were enrolled in the study, with 16 patients monitored using both written methods and the OITcontrol app (ie, the OITcontrol group) and 14 patients monitored solely through a written paper diary (ie, the PaperPRO group). A comparable distribution was observed concerning sex, age, basal characteristics (bronchial asthma/previous anaphylaxis with OIT food), the allergen used in OIT, premedication, and sensitization profile (Table 1).

No significant differences (*P*=.07) were observed in terms of follow-up time between PaperPRO patients (median 146.5 days, Q1-Q3 98-213 days) and OITcontrol patients (median 196.5 days, Q1-Q3 147.5-336.5 days). However, it is worth noting that 5/14 (36%) PaperPRO patients and 13/16 (81%) OITcontrol patients (*P*=.01) underwent OIT treatment during the COVID-19 pandemic. In fact, a similar number of hospital dose increases were observed between PaperPRO patients (mean 9.9, SD 6.4) and OITcontrol patients (mean 14, SD 9.5; *P*=.18). Likewise, the number of home OIT days was comparable between the PaperPRO group (median 128, Q1-Q3 89-192) and the OITcontrol group (median 167.5, Q1-Q3 135-287.5; *P*=.11).

**Table 1:** Baseline characteristics of the sample.

Characteristics				PaperPRO group (n=14)		OITcontrol group (n=16)		*P* value
Female, n (%)				5 (36)		10 (63)		.14
Age (years), mean (SD)				6.9 (1.9)		7.6 (3.7)		.75
Asthma, n (%)				5 (36)		7 (44)		.65
Previous anaphylaxis with OIT^a^ allergen, n (%)				7 (50)		13 (81)		.08
OIT with egg, n (%)				8 (57)		8 (50)		.70
OIT with milk, n (%)				6 (43)		8 (50)		.70
Antihistaminic premedication, n (%)				0 (0)		4 (25)		.07
Omalizumab premedication, n (%)				1 (7)		1 (6)		.92
Total immunoglobulin E value (kU/L), median (Q1-Q3^b^)				494.5 (120-858)		258 (87-931)		.57
**In egg OIT patients**								
	OIT with egg, n (%)		8 (57)		8 (50)		.70	
	**Specific immunoglobulin E (kUA/L), median (Q1-Q3)**							
		Egg white	7.7 (2.2-13.8)		9 (5.7-16.2)		.56	
		Egg yolk	1.9 (0.5-4.4)		3 (1.1-7.6)		.40	
		Ovalbumin	4 (0.6-8.8)		4.9 (1.4-9)		.67	
		Ovomucoid	4.1 (0.5-14.1)		8.1 (4.5-17.3)		.21	
	**Prick test diameter (mm), median (Q1-Q3)**							
		Egg white	7 (3-9.7)		8 (6.5-11)		.46	
		Egg yolk	5 (2.2-7.5)		6.5 (3-8)		.67	
		Ovalbumin	6.2 (2.7-10)		9 (6-10)		.53	
		Ovomucoid	5.5 (4.2-9.7)		9.5 (7-15)		.19	
**In milk OIT patients**								
	OIT with milk, n (%)		6 (43)		8 (50)		.70	
	**Specific immunoglobulin E (kUA/L), median (Q1-Q3)**							
		Milk	5.9 (3.9-7.8)		8.6 (3.8-19.8)		.70	
		Alpha-lactalbumin	1.1 (0.4-3.2)		0.4 (0.1-9.8)		.30	
		Beta-lactoglobulin	0.7 (0.4-1.4)		0.5 (0.2-0.9)		.52	
		Casein	3.7 (1.6-9.4)		2.8 (0.8-13.6)		.56	
	**Prick test diameter (mm), median (Q1-Q3)**							
		Milk	4.7 (3-5)		4.7 (3.5-10.2)		.56	
		Alpha-lactalbumin	5.5 (4.5-6)		3 (0-7)		.49	
		Beta-lactoglobulin	6.7 (5.5-9.5)		7 (3.5-9.2)		.43	
		Casein	7.5 (3-9)		3.7 (1-12)		.56	

^a^OIT: oral immunotherapy.

^b^Q1-Q3: first quartile-third quartile.

### OIT Adverse Reactions

PaperPRO patients experienced 5 hospital reactions, while OITcontrol patients experienced 19 hospital reactions. Table 2 summarizes hospital reactions. In the PaperPRO group, the 5 hospital reactions were experienced by 5 different patients (1 reaction per patient), whereas in the active group, the 19 reactions were experienced by only 3 patients (the first patient had 1 reaction, the second had 3 reactions, and the third had 15 reactions). No differences were observed regarding the number of hospital reactions per patient, the number of hospital reactions per hospital dose given, the type of reactions, or the severity of the reactions between both groups of patients.

Concerning home reactions, PaperPRO patients reported 56 home reactions, while OITcontrol patients reported 97 home reactions (*P*=.70). Table 3 summarizes home reactions. More than one-half of all patients included in the study (19/30, 63%) experienced a home reaction. Globally, only moderate home reactions were more frequently reported by PaperPRO patients than OITcontrol patients (*P*=.047). However, no differences were observed regarding the specific type of reaction between both groups of patients.

**Table 2:** Hospital reactions.

Reactions						PaperPRO group (n=14)			OITcontrol group (n=16)			*P* value			
Hospital reactions, n						5			19			.45			
Hospital reaction/hospital visit, median (Q1-Q3^a^)						0 (0-0.1)			0 (0-0)			.18			
Patients with hospital reactions, n (%)						5 (36)			3 (19)			.29			
Hospital reaction/patient, median (Q1-Q3)						0 (0-1)			0 (0-0)			.45			
**Type of hospital reaction**															
	**Mild reactions, reactions (affected patients), n**				2 (2)			5 (2)			>.99				
			Mild OAS^b^	0 (0)			1 (1)			.35					
			Relevant OAS (lip edema/perioral urticaria)	1 (1)			2 (1)			.96					
			Facial urticaria/angioedema	1 (1)			0 (0)			.29					
			Mild gastrointestinal symptoms	0 (0)			2 (1)			.35					
	**Moderate reactions, reactions (affected patients), n**				3 (3)			7 (1)			.31				
		Acute generalized urticaria		1 (1)			1 (1)			.92					
		Rhinoconjunctivitis		2 (2)			6 (1)			.52					
	**Severe reactions, reactions (affected patients), n**				0 (0)			7 (2)			.18				
		Severe gastrointestinal symptoms		0 (0)			1 (1)			.35					
		Oropharyngeal discomfort		0 (0)			3 (1)			.35					
		Bronchospasm		0 (0)			3 (1)			.35					
		Anaphylaxis		0 (0)			0 (0)			>.99					
		Anaphylactic shock		0 (0)			0 (0)			>.99					

^a^Q1-Q3, first quartile-third quartile.

^b^OAS: oral allergy syndrome.

**Table 3:** Reported home reactions.

Reactions reported			PaperPRO group (n=14)		OITcontrol group (n=16)		*P* value
Home reactions, n			56		97		.70
Home reactions/home doses, median (Q1-Q3^a^)			0 (0-0.06)		0 (0-0.07)		.76
Patients with home reactions, n (%)			9 (64)		10 (63)		.12
Home reactions/patient, median (Q1-Q3)			3 (0-5)		2.5 (0-11.5)		.70
**Mild reactions, reactions (affected patients), n**			38 (6)		73 (8)		.53
	Mild OAS^b^	12 (3)		37 (7)		.17	
	Relevant OAS (lip edema/perioral urticaria)	11 (3)		6 (4)		.95	
	Facial urticaria/angioedema	7 (3)		3 (3)		.72	
	Mild gastrointestinal symptoms	8 (3)		27 (5)		.47	
**Moderate reactions, reactions (affected patients), n**			13 (6)		2 (2)		.047
	Acute generalized urticaria	3 (3)		1 (1)		.23	
	Rhinoconjunctivitis	10 (4)		1 (1)		.09	
**Severe reactions, reactions (affected patients), n**			5 (3)		22 (6)		.21
	Severe gastrointestinal symptoms	1 (1)		8 (3)		.32	
	Oropharyngeal discomfort	3 (2)		9 (4)		.42	
	Bronchospasm	1 (1)		0 (0)		.28	
	Anaphylaxis	0 (0)		5 (3)		.09	
	Anaphylactic shock	0 (0)		0 (0)		>.99	

^a^Q1-Q3: first quartile-third quartile.

^b^OAS: oral allergy syndrome.

### Home Data Recording

In the OITcontrol group, 81% (13/16) of patients used the OITcontrol app, while in the PaperPRO group, 71% (10/14) used the written diary (*P*=.53). As mentioned previously, patients in the OITcontrol group were advised to record daily taken allergen doses and home reactions using both methods: the written paper and the app. Following these recommendations, 38% (6/16) of OITcontrol patients used both methods. One active patient used only the written diary without using the OITcontrol app. Interestingly, none of the patients collected all daily dose intakes, regardless of the monitoring method used.

**Figure 3:**
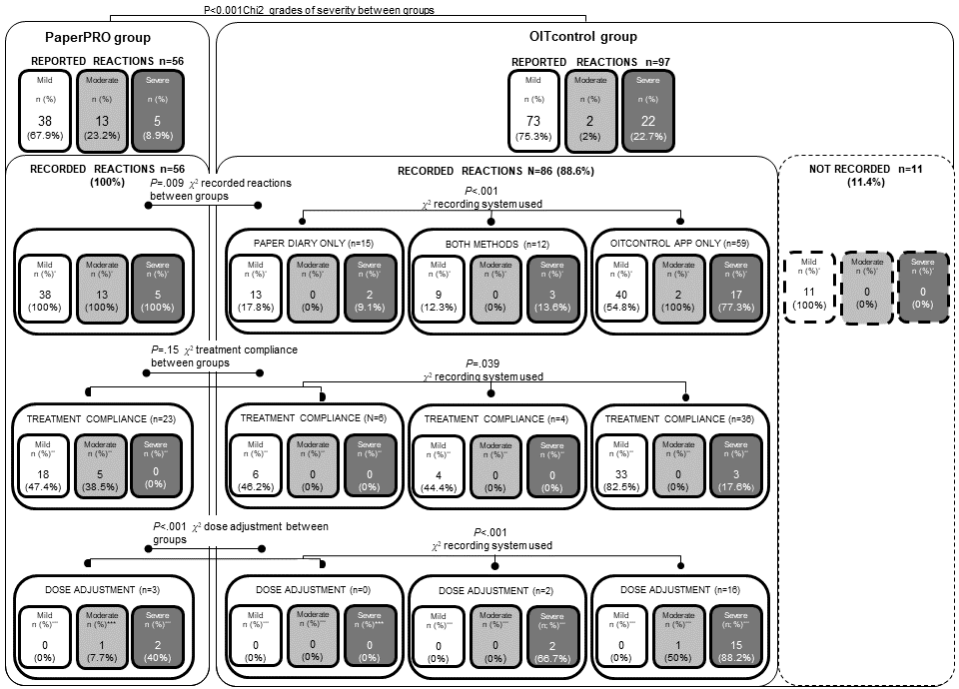
Summary of reported and recorded home reactions in both study groups, including the number of reactions documented, the treatment compliance and the dose adjustment performed after a moderate or severe reaction in recorded reactions through written diary, OITcontrol® app, or a combination of both monitoring systems. * Percentage of recorded reactions among reported reactions for each severity grade in each group. ** Percentage of reactions that followed indicated treatment among recorded reactions for each severity grade in each group. *** Percentage of reactions that follow dose adjustment after a moderate-severe reaction among recorded reactions for each severity grade in each group.

When analyzing reported reactions, every home reaction experienced in the PaperPRO group was recorded in the written diary (56/56, 100%), while 89% (86/97 reactions) of the home reactions experienced in the OITcontrol group were recorded (*P*=.009). Active patients preferred using only the app (59/86, 69%) rather than the written diary (15/86, 17%) or both methods (12/86, 14%) to record home reactions (*P*<.001). These data are summarized in Figure 3. Every reaction recorded by the OITcontrol group in both recording methods, written diary and OITcontrol app, was documented using the same description regarding allergen dose, type of symptoms, and administered treatment.

### App Assistance Evaluation

Treatment compliance in home-recorded reactions was analyzed between both groups and the monitoring method used. The indicated treatment was followed in 23 of the 56 (41%) recorded reactions in the PaperPRO group and in 46 of the 86 (53%) home-recorded reactions in the OITcontrol group (*P*=.15). Analyzing the monitoring system used, treatment was observed to be followed more frequently (*P*=.04) in reactions recorded only in the app (36/59, 61%) than in the written diary (29/71, 41%) or both systems (4/12, 33%; Figure 3). In general, treatment compliance was observed more frequently (*P*<.001) in mild reactions (61/100, 61%) than in moderate (5/15, 33%) and severe reactions (3/27, 11%). However, it is worth noting that in recorded mild reactions, treatment compliance was quite high, possibly because no treatment was indicated for the frequently reported mild OAS (every recorded mild OAS was correctly managed in both groups: 12 in the PaperPRO group and 31 in the OITcontrol group). In fact, mild reactions excluding mild OAS (18/57, 32%), moderate (5/15, 33%), and severe reactions (3/27, 11%) followed treatment prescription correctly in similar rates (*P*=.11). Interestingly, in most of the reactions where prescribed treatment was not followed, the common attitude among patients was not to apply any treatment, which was consistent across both groups. Detailed data are provided in Tables 4-6.

Dose adjustments after a moderate-severe reaction in home-recorded reactions were analyzed between both groups and the monitoring method used. Among the 18 doses that should have been adjusted after a moderate-severe reaction in the PaperPRO group, only 3 (17%) were adjusted. By contrast, among the 24 recorded doses requiring adjustment after a moderate-severe reaction in the OITcontrol group, 18 (75%) were correctly adjusted (*P*<.001). In general, dose adjustment was more frequently performed (*P*<.001) in those reactions recorded only in the app (16/19, 84%) than in those recorded in the written diary (3/20, 15%) or in both methods (2/3, 67%). The severity of reactions was associated with better compliance, as adjustments were made after severe reactions (19/27, 70%) more frequently (*P*<.001) than after moderate reactions (2/15, 13%). Data are summarized in Figure 3.

**Table 4:** Comparison of the adequacy of the treatment applied in recorded reactions of written diary users from both groups of patients.^a^

Written diary (indicated treatment)	Treatment applied					
	None	AH^b^	AH and CST^c^	EPI^d^	EPI and BD^e^	BD
None	*14* ^f^	0	0	0	0	0
AH	33	*14*	0	0	0	0
AH and CST	0	2	*1*	0	0	0
EPI	6	0	0	*0*	0	0
EPI and BD	0	0	0	0	*0*	1

^a^The number of reactions among both groups of patients is represented by comparing the treatment applied and indicated treatment in written diary-recorded reactions.

^b^AH: antihistamine.

^c^CST: corticosteroid.

^d^EPI: epinephrine.

^e^BD: bronchodilator.

^f^Italicized values indicate the number of patients that followed the prescribed treatment correctly.

**Table 5:** Comparison of the adequacy of the treatment applied in recorded reactions of OITcontrol app users from both groups of patients.^a^

Written diary (indicated treatment)	Treatment applied					
	None	AH^b^	AH and CST^c^	EPI^d^	EPI and BD^e^	BD
None	*27* ^f^	0	0	0	0	0
AH	8	*6*	0	0	0	0
AH and CST	0	1	*0*	0	0	0
EPI	10	3	1	*3*	0	0
EPI and BD	0	0	0	0	*0*	0

^a^The number of reactions among both groups of patients is represented by comparing the treatment applied and indicated treatment in the OITcontrol app–recorded reactions.

^b^AH: antihistamine.

^c^CST: corticosteroid.

^d^EPI: epinephrine.

^e^BD: bronchodilator.

^f^Italicized values indicate the number of patients that followed the prescribed treatment correctly.

**Table 6:** Comparison of the adequacy of the treatment applied in recorded reactions of OITcontrol app and written diary users from both groups of patients.^a^

Written diary and OITcontrol (indicated treatment)	Treatment applied					
	None	AH^b^	AH and CST^c^	EPI^d^	EPI and BD^e^	BD
None	*2* ^f^	0	0	0	0	0
AH	5	*2*	0	0	0	0
AH and CST	0	0	*0*	0	0	0
EPI	0	2	1	*0*	0	0
EPI and BD	0	0	0	0	*0*	0

^a^The number of reactions among both groups of patients is represented by comparing the treatment applied and indicated in both OITcontrol app– and written paper–recorded reactions.

^b^AH: antihistamine.

^c^CST: corticosteroid.

^d^EPI: epinephrine.

^e^BD: bronchodilator.

^f^Italicized values indicate the number of patients that followed the prescribed treatment correctly.

### Users’ Satisfaction Questionnaire

A total of 15 users answered the questionnaire in the first week, and 11 responded in the fourth week. Among the 15 users answering in the first week of app use, 7/15 (47%) were females, with most aged between 35 and 44 years (8/15, 53%); 5/15 (33%) were between 45 and 54 years and 2/15 (13%) were between 25 and 34 years; 9/15 (60%) of them reported very frequent use of a smartphone (1=no use to 5=very frequent use: 3/15, 20%, rated the use 4/5; 2/15, 13%, rated the use 3/5; and 1/15, 7%, rated the use 2/5). In general, the app received positive ratings, being considered easy to use in most functions and screens, with suitable text. However, there were suggestions that error messages could be clearer. The questionnaire results are summarized in Tables 7-9.

**Table 7:** Results of the usability questionnaire after 1 week and 4 weeks of use of the OITcontrol app: general opinions.

Questions and rating			Results at 1 week of use		Results at 4 weeks of use
**OITcontrol app is**					
	1=terrible to 5=wonderful, mean (SD)	3.9 (0.7)		4.3 (0.6)	
	1=frustrating to 5=easy, mean (SD)	4.1 (0.7)		4.3 (0.6)	
	1=boring to 5=exciting, mean (SD)	3.6 (0.8)		3.8 (0.9)	
	1=difficult to 5=easy, median (Q1-Q3^a^)	5 (4-5)		5 (4-5)	
	1=too slow to 5=too fast, mean (SD)	3.3 (1)		3.2 (0.9)	
	1=unreliable to 5=highly reliable, median (Q1-Q3)	3.9 (3.9-4.4)		3.9 (2.8-4.4)	
	1=noisy to 5=noiseless, mean (SD)	4.4 (0.6)		4.7 (0.5)	

^a^Q1-Q3: first quartile-third quartile.

**Table 8:** Results of the usability questionnaire after 1 week and 4 weeks of use of the OITcontrol app: opinion about how easy/difficult is to use different functions.

Questions	Rating	Results at 1 week of use	Results at 4 weeks of use
Know I should take the dose	1=difficult to 5=easy, median (Q1-Q3^a^)	5 (5-5)	5 (5-5)
Know how to take the dose	1=difficult to 5=easy, median (Q1-Q3)	5 (4-5)	5 (4-5)
Know indications after reaction	1=difficult to 5=easy, mean (SD)	4.6 (0.5)	4.6 (0.5)
Record the dose intake and its additional information	1=difficult to 5=easy, mean (SD)	4.6 (0.5)	4.8 (0.4)
Receive the alarm at the dose intake time	1=difficult to 5=easy, mean (SD)	3.9 (1)	4 (1.2)
Consult past dose intake record	1=difficult to 5=easy, median (Q1-Q3)	5 (4-5)	5 (4-5)
Consult the next hospital visit	1=difficult to 5=easy, median (Q1-Q3)	5 (4-5)	5 (4-5)
To correct mistakes	1=difficult to 5=easy, mean (SD)	3.5 (0.9)	3.5 (1.2)

^a^Q1-Q3: first quartile-third quartile.

**Table 9:** Results of the usability questionnaire after 1 week and 4 weeks of use of the OITcontrol app: opinions about text and screens.

Questions	Rating	Results at 1 week of use	Results at 4 weeks of use
The texts on the screen are...difficult or easy to read?	1=difficult to 9=easy, median (Q1-Q3^a^)	8 (7-9)	8 (7-9)
Is the information highlighted helpful?	1=absolutely not to 9=of course yes, median (Q1-Q3)	8 (7-9)	8 (7-9)
Is the transition from one screen/information to another confusing or clear?	1=confuse to 9=clear, median (Q1-Q3)	8 (7-8)	7 (6-8)
Does the use of terms...encourage or discourage its use?	1=discourage to 9=encourage, mean (SD)	5.9 (2.3)	5.8 (2.5)
Does the use of terms encourage or discourage learning?	1=discourage to 9=encourage, mean (SD)	6.2 (2.5)	6.3 (2.1)
Error messages...are they confusing or clear?	1=confuse to 9=clear, mean (SD)	6.6 (1.7)	5.5 (2.5)
The messages that appear on the screen...Are they difficult or simple?	1=difficult to 9=simple, mean (SD)	7.9 (1.1)	8.2 (0.9)

^a^Q1-Q3: first quartile-third quartile.

## Discussion

### Principal Findings

This study demonstrates that OITcontrol, a patient advisor app incorporating medical algorithms, goes beyond serving as an electronic report and is an effective method for monitoring home OIT. Moreover, our findings suggest that OITcontrol emerges as an appealing method for overseeing OIT treatments, as it has been predominantly used by the active group. Additionally, instructions provided by the app have been adhered to more consistently than the written indications regarding treatment and dose adjustments following a reaction.

eHealth technology has seen widespread adoption in recent years, particularly in the context of respiratory allergy [31-33]. Conversely, the application of eHealth technology in food allergy has primarily focused on the development of mobile apps designed to complement patient care. These apps often provide features such as allergen-free product searches, meal planners, or tools for locating allergy-adapted restaurants [34,35]. OITcontrol aligns with the objectives of eHealth apps, serving not only the beneficial purposes for patients with allergies but also catering to the needs of clinicians and researchers [36]. It exemplifies the use of health informatics by automating physician orders [37].

Previous reports have indicated that as few as 20% of patients are genuinely compliant with paper-based diaries [38]. In our sample, reporting compliance was remarkably high. PaperPRO patients exhibited perfect adherence in recording home reactions, surpassing the OITcontrol group. In the OITcontrol group, patients displayed a preference for recording home reactions within the app. This observation may be due to the control group’s potentially better performance when using only 1 monitoring system, as opposed to the active group using 2 systems. Alternatively, it could be indicative of underreporting of home reactions by the control group, possibly trivializing or forgetting to report reactions when using standard methods compared with having an additional monitoring intervention.

Indeed, a previous electronic web-based reporting system implemented for OIT, which focused on dose and home reactions reporting, demonstrated higher adherence than that observed in our sample. However, the rate of reported home reactions was quite similar to our data [39]. Nevertheless, Nachshon et al [39] highlighted some limitations of this monitoring web-based system, including challenges related to the patient’s description of reactions. In this regard, OITcontrol provides a tabulated selection of reactions rather than an open-ended description box. It appears that these predefined reactions are effectively described, as treatment compliance and dose adjustment after a reaction were more successful, particularly for those reactions recorded in the app.

Home reactions documented in the OITcontrol app were more consistently treated correctly compared with those recorded in the written diary, despite the fact that treatment compliance was notably low, particularly among patients experiencing moderate and severe reactions. It is worth noting that epinephrine is underused in cases of anaphylaxis, even among well-informed and trained parents familiar with the use and indications of autoinjectors. This could be attributed to reasons such as the unavailability of the autoinjector, difficulty in recognizing anaphylaxis, and concerns about potential adverse effects [40-45]. In our limited sample, patients who required self-injectable epinephrine rarely used it, irrespective of whether they followed written or electronic recommendations. However, the correct treatment in mild reactions was more frequently adhered to. Further, a larger sample of patients is needed to assess whether the OITcontrol app could enhance treatment compliance for home reactions and contribute to adjusting home doses after moderate-severe reactions. Our data, albeit based on a limited number of reactions, suggest that OITcontrol app recommendations regarding dose adjustment were followed more consistently than written recommendations.

### Conclusions

In conclusion, the OITcontrol app appears to enhance treatment and dose adjustment compliance in home reactions, although further studies are needed to confirm the efficacy of the app in this regard. As a monitoring system, the OITcontrol app is deemed a suitable method in OIT treatment for recording daily dose intakes and home reactions during the buildup phase.

## Appendix

Multimedia Appendix 1 Supplementary Table S1: Clinical description, classification, and treatment of reactions.

## Abbreviations

AH: antihistamine
BD: bronchodilator
CST: corticosteroid
EPI: epinephrine
IgE: immunoglobulin E
OAS: oral allergy syndrome
OIT: oral immunotherapy
