# Supplementary material for: Mobile App/Web Platform for Monitoring Food Oral Immunotherapy in Children: Longitudinal Clinical Validation Study
Source: JMIR Pediatr Parent. 2024 Mar 13;7:e54163. doi: 10.2196/54163 (PMC10973957; doi:10.2196/54163)
Supplement: Multimedia Appendix 1 [file pediatrics_v7i1e54163_app1.docx]

**Table S1.** Clinical description, classification, and treatment of reactions.

| Reaction | Image | Reaction description for patient | Treatment | Recommendations after reaction | Severity |
| --- | --- | --- | --- | --- | --- |
| Mild OAS^a^ | 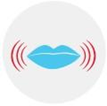 | Mild itching in the mouth | Drink water and stay calm | If it lasts >15 minutes, take 1 dose of antihistamine. If other symptoms appear, treatment should be reconsidered. | Mild |
| Relevant OAS (lip edema/perioral urticaria) | 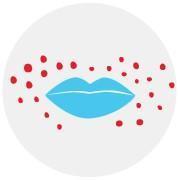 | Itching of the mouth/throat, hives around the mouth, or slight swelling of the lips | Antihistamine | If it does not improve in 1-2 hours, go to the emergency room. If other symptoms appear, treatment should be reconsidered. | Mild |
| Facial urticaria/angioedema | 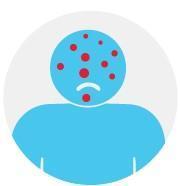 | Itching or redness of the face, hives on the face, or swelling of lips or eyelids | Antihistamine | If it does not improve in 1-2 hours, go to the emergency room. If other symptoms appear, treatment should be reconsidered. | Mild |
| Mild gastrointestinal symptoms | 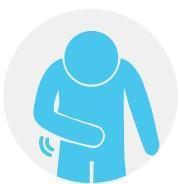 | Mild abdominal pain/discomfort or isolated vomiting/nausea/diarrhea | Antihistamine | If the pain lasts more than 45-60 minutes, administer intramuscular adrenaline and go immediately to the emergency room. | Mild |
| Acute generalized urticaria | 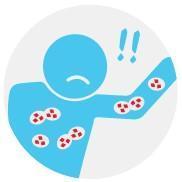 | Generalized itching, redness, or hives over the body and swelling of the hands or feet | Antihistamine and corticosteroids | After taking the medication, you should go immediately to the emergency room. If other symptoms appear, treatment should be reconsidered. | Moderate |
| Rhinoconjunctivitis | 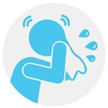 | Nasal congestion, tearing, itchy eyes, red eyes, sneezing, or runny nose | Antihistamine | If other symptoms appear, treatment should be reconsidered. | Moderate |
| Severe gastrointestinal symptoms | 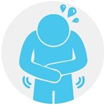 | Severe abdominal pain, nausea, vomiting, or ongoing diarrhea | Epinephrine | If abdominal pain lasts >45-60 minutes or vomiting/diarrhea persists, administer intramuscular adrenaline and go immediately to the emergency department. If other symptoms appear, treatment should be reconsidered. | Severe |
| Oropharyngeal discomfort | 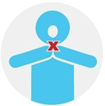 | Throat lump/oppression, salivation/protrusion of the tongue, and voice changes | Epinephrine | You should go immediately to the emergency room. If there is no improvement within 5-15 minutes, administer the second adrenaline device again. Antihistamine and corticosteroids should be used as additional treatment. If other symptoms appear, the treatment should be reconsidered. | Severe |
| Bronchospasm | 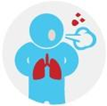 | Chest tightness, cough, shortness of breath, and whistling in the chest | Epinephrine and salbutamol | You should go immediately to the emergency room. If there is no improvement within 5-15 minutes, administer the second adrenaline device and bronchodilator again. | Severe |
| Anaphylactic shock | 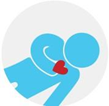 | Pallor, weakness, loss of consciousness, and sensation of imminent death | Epinephrine | You should go immediately to the emergency room. If there is no improvement within 5-15 minutes, administer the second adrenaline device again. | Severe |

^a^OAS: oral allergy syndrome.
